# Speech and Language Changes During Rapid Eye Movement (REM) Sleep with Potential Diagnostic Markers: A Systematic Review

**DOI:** 10.3390/brainsci16020216

**Published:** 2026-02-11

**Authors:** Maria Pagano, Francesco Corallo, Anna Anselmo, Davide Cardile, Rosaria De Luca, Angelo Quartarone, Rocco Salvatore Calabrò, Irene Cappadona

**Affiliations:** IRCCS Centro Neurolesi Bonino-Pulejo, Via Palermo, S.S. 113, C.da Casazza, 98124 Messina, Italy; maria.pagano@irccsme.it (M.P.);

**Keywords:** REM sleep behavior disorder (RBD), vocal biomarker, speech analysis, neurodegenerative disease

## Abstract

**Background:** Rapid Eye Movement (REM) sleep behavior disorder (RBD) is a parasomnia resulting from degeneration of pontine and medullary circuits responsible for muscle atonia during REM sleep, leading to dream-enactment behaviors and vocalizations. It is strongly linked to α-synucleinopathies, particularly Parkinson’s disease. Current biomarkers such as neurophysiological measures and imaging support diagnosis and monitoring, but remain invasive or costly. **Aim:** This study aims to evaluate vocal and speech alterations as exploratory, non-validated candidate biomarkers of REM sleep behavior disorder. **Methods:** A systematic review was conducted according to PRISMA 2020 guidelines. PubMed, IEEE Digital Library Web of Science, Embase and the Cochrane Library were systematically searched for studies published from database inception to November 2025, as preregistered on the Open Science Framework. Studies were selected through a multi-step screening process and underwent qualitative quality assessment. **Results:** Twelve studies met inclusion criteria. Individuals with RBD exhibited abnormal nocturnal vocalizations and early lexical, syntactic, and narrative disruptions despite preserved perceptual speech. Quantitative analyses identified consistent deficits in prosody, phonation stability, timing, and articulation, with significant group differences and diagnostic accuracy up to 96% sensitivity. Multilingual cohorts demonstrated progression over time, while digital phenotyping detected emerging Parkinsonian signs with AUC > 0.70. **Conclusions:** Speech and vocal abnormalities in iRBD reflect early neurodegenerative changes and show promising but still exploratory diagnostic and prognostic potential. Integrating vocal markers with established biomarkers may enhance early detection; however, further research is required to validate a reliable and reproducible vocal signature of prodromal synucleinopathies.

## 1. Introduction

Sleep is a fundamental biological process that is essential for physical health, cognitive performance, emotional regulation, and overall quality of [1]. In adults, current consensus guidelines recommend an average sleep duration of approximately seven to nine hours per night, as both chronic sleep deprivation and excessive sleep are associated with increased morbidity and mortality [2,3]. Adequate sleep quantity and quality are closely linked to pain perception, immune function, cardiovascular health, and mental well-being. Disturbed sleep has been shown to exacerbate chronic pain, impair emotional resilience, and reduce cognitive efficiency, while restorative sleep supports learning, memory consolidation, and adaptive stress responses [4,5]. From a broader clinical perspective, sleep disorders often coexist with systemic and neurological conditions. Obstructive sleep apnea (OSA), temporomandibular disorders (TMD), chronic pain syndromes, mood disorders, and neurodegenerative diseases often interact bidirectionally with sleep, forming a complex network of behavioral, physiological, and neurobiological influences. The field of behavioral sleep medicine emphasizes the role of sleep-related behaviors, circadian regulation, and psychological factors in the development and maintenance of sleep disorders, highlighting sleep as a modifiable determinant of overall health rather than a passive state [6,7,8,9]. REM sleep is involved in emotional processing, memory integration, autonomic regulation, and brain plasticity. Disruption of REM sleep mechanisms may therefore have profound consequences for both neural integrity and behavioral regulation [10]. In addition, it is important to recognize that sleep disturbances are not unique to neurodegenerative disorders but are also highly prevalent in several congenital and craniofacial conditions. Syndromes such as Treacher–Collins syndrome, cleidocranial dysplasia, and Goldenhar syndrome are frequently associated with structural airway abnormalities, altered craniofacial morphology, and neuromuscular dysfunction, predisposing affected individuals to sleep-disordered breathing, fragmented sleep, and abnormal sleep-related vocalizations. Although the pathophysiological mechanisms underlying sleep disturbances in these congenital conditions differ from those of RBD, their inclusion underscores the broader relevance of sleep assessment and highlights the necessity of differential diagnostic considerations when interpreting nocturnal motor or vocal behaviors [11,12,13].

Rapid Eye Movement (REM) Sleep Behavior Disorder (RBD) is a parasomnia marked by the failure of the physiological muscle atonia that typically accompanies rapid eye movement sleep [14]. Under normal conditions, the skeletal musculature is actively inhibited during REM sleep by neural circuits in the pontine tegmentum, preventing the physical enactment of dreams. When this mechanism fails, patients may reproduce dream content through complex and sometimes violent movements and a wide range of vocalizations, including shouting, talking, laughing, or crying [15]. These episodes usually occur in the latter part of the night, when REM sleep predominates. Video-polysomnography (PSG) demonstrates REM sleep without atonia (RSWA), the electrophysiological hallmark of RBD and the key diagnostic criterion described in the International Classification of Sleep Disorders-Third Edition, Text Revision (ICSD-3-TR) [16]. It is important to distinguish RBD from other parasomnias associated with motor behaviors during sleep, particularly non-REM parasomnias such as sleepwalking [17]. While both conditions may involve complex and sometimes injurious movements, sleepwalking typically arises from slow-wave sleep, occurs predominantly in the first third of the night, and is characterized by absent or minimal dream recall. In contrast, RBD episodes emerge from REM sleep, are closely linked to vivid and often violent dream mentation, and are associated with preserved consciousness upon awakening. These differences reflect distinct underlying neurophysiological mechanisms and have important diagnostic and prognostic implications [18].

From a neurobiological perspective, RBD originates from dysfunction within the brainstem networks responsible for generating REM sleep and suppressing motor output [19]. The sublaterodorsal nucleus (SLD) in the pons and the magnocellular reticular formation in the medulla are essential for the maintenance of muscle atonia during REM sleep [20]. These structures project inhibitory signals through glycinergic and GABAergic interneurons to spinal and cranial motoneurons [6]. When these inhibitory pathways are disrupted, motor activity becomes disinhibited, leading to dream enactment behaviors. Experimental studies on lesions and animal models have shown that selective damage or functional inhibition of the sublaterodorsal nucleus and ventromedial medulla abolishes REM sleep atonia and induces complex motor and vocal behaviors that are very similar to human RBD [21,22,23]. In humans, post-mortem analyses and neuroimaging confirmed degenerative changes in pontine and medullary nuclei, particularly within the locus coeruleus, subcoeruleus complex, and pedunculopontine tegmental nucleus. These regions are rich in monoaminergic and cholinergic neurons that regulate REM-related motor inhibition and state generation [24]. Their degeneration leads to the disinhibition of descending motor pathways and to the emergence of the motor and vocal phenomena typical of RBD. These same nuclei constitute early sites of α-synuclein deposition, supporting the view that RBD represents a prodromal stage of α-synucleinopathy [25].

Functional neuroimaging has shown that RBD also involves widespread dysfunction of cortico-subcortical networks, including the basal ganglia, prefrontal cortex, anterior cingulate cortex, amygdala, and hypothalamus [26]. Decreased connectivity between the striatum and frontal regions and altered limbic activation have been described during REM sleep, which may explain both the emotional vividness of the dreams and the frequent autonomic activation that accompanies the episodes [27]. Degeneration of monoaminergic neurons in the locus coeruleus and the dorsal raphe nucleus reduces the inhibitory tone within the motor system, further contributing to RSWA. The progressive involvement of dopaminergic neurons in the substantia nigra pars compacta provides a direct link between RBD and the later development of Parkinson’s disease and other synucleinopathies [28].

Clinically, RBD is characterized by vigorous dream enactment behaviors and vocalizations that may result in injury to the patient or bed partner. The disorder may appear as an idiopathic condition or as a manifestation secondary to neurological, structural, or pharmacological causes [29]. The idiopathic form, known as iRBD, is now considered one of the most reliable early markers of neurodegenerative disease, particularly of α-synucleinopathies such as Parkinson’s disease, dementia with Lewy bodies, and multiple system atrophy [30]. Longitudinal cohort studies indicate that between 80 and 90 percent of patients with iRBD develop one of these disorders within ten to fifteen years [25,31]. The estimated prevalence in the general population is approximately 0.5 to 1 percent, with a clear male predominance and peak incidence beyond the age of fifty [30]. Patients with iRBD frequently present with hyposmia, constipation, orthostatic hypotension, mild executive dysfunction, and emotional dysregulation, indicating early involvement of dopaminergic, limbic, and autonomic circuits [32].

According to the ICSD-3-TR, the diagnosis of RBD requires the occurrence of repeated episodes of complex motor or vocal behaviors associated with vivid or violent dreams, polysomnographic confirmation of REM sleep without atonia, exclusion of other potential causes, and evidence of clinically significant consequences such as injuries or disturbed sleep. Video-polysomnography remains the gold standard for confirmation, although bed-partner reports of dream enactment and vocalizations are highly sensitive for clinical screening [33].

In recent years, increasing attention has been directed toward speech- and voice-derived measures as potential biomarkers for the detection and monitoring of RBD. According to international consensus definitions, a biomarker is an objectively measured characteristic that reflects normal biological processes, pathogenic processes, or responses to an intervention, and that demonstrates adequate reliability, validity, and clinical relevance [34]. Within this framework, most vocal and linguistic measures investigated in RBD should currently be regarded as exploratory speech or vocal markers rather than established biomarkers, as their diagnostic thresholds, specificity, and longitudinal stability remain under investigation [35]. Nocturnal vocalizations are an intrinsic component of the disorder, representing direct activation of laryngeal and respiratory musculature due to the loss of REM-related inhibition [33]. Such voice alterations are thought to arise from dopaminergic and bulbar dysfunction within neural circuits controlling phonation and respiration [36]. Acoustic analyses focusing on parameters such as jitter, shimmer, harmonic-to-noise ratio, and fundamental frequency variability have identified distinctive patterns in iRBD that correlate with both disease severity and neurodegenerative risk [37]. Because these methods are noninvasive, inexpensive, and suitable for remote digital acquisition, they provide an ideal platform for longitudinal monitoring of disease evolution and treatment response. The integration of voice analysis with established physiological and imaging measures may, in the future, enable earlier diagnosis, better risk stratification, and personalized follow-up in patients at high risk for Parkinson’s disease and related disorders. However, before language- or voice-based measurements can be considered validated biomarkers, further large-scale longitudinal studies, external validation, and standardization of acquisition and analysis protocols are needed. This approach, facilitated by modern diagnostic technologies [38,39], supports the broader vision of predictive and preventive neurology, which aims to identify and modify neurodegenerative processes before clinical onset.

In this context, the present systematic review aims to evaluate the existing evidence on vocal and speech alterations observed during both sleep and wakefulness as exploratory, noninvasive candidate biomarkers of REM Sleep Behavior Disorder (RBD).

The specific objectives are:

(i) to systematically identify and synthesize studies investigating speech, voice, and language alterations in individuals with RBD;
(ii) to critically assess the methodological quality and risk of bias of the included studies;
(iii) to analyze and compare the main acoustic, prosodic, and linguistic findings across studies; and
(iv) to evaluate the potential diagnostic and prognostic relevance of these measures in relation to neurodegenerative risk and phenoconversion.

We hypothesize that individuals with RBD exhibit detectable vocal and speech abnormalities during both sleep and wakefulness, reflecting early dysfunction of brainstem and cortico-subcortical networks implicated in α-synucleinopathies.

We further hypothesize that these alterations, although currently exploratory, may precede overt motor symptoms and hold potential value for early detection and longitudinal disease monitoring, pending further validation.

The manuscript is structured as follows: Section 2 describes the materials and methods; Section 3 presents the synthesis of the results of the included studies, including quality assessment; Section 4 discusses the findings in relation to pathophysiology and clinical implications, outlines strengths, limitations, and future research directions; and Section 5 summarizes the conclusions.

## 2. Materials and Methods

A systematic review of the literature was conducted to examine acoustic, prosodic, and linguistic features associated with REM Sleep Behavior Disorder (RBD). The review followed the Preferred Reporting Items for Systematic Reviews and Meta-Analyses (PRISMA) 2020 guidelines [40]. The completed PRISMA checklist is provided in the Supplementary Materials. The review protocol was prospectively registered on OSF.io (https://doi.org/10.17605/OSF.IO/QEJRH; accessed on 2 December 2025).

### 2.1. Search Strategy and Inclusion Criteria

A comprehensive literature search was conducted in the following electronic databases: PubMed, IEEE Digital Library, Web of Science, Embase and Cochrane Library. The search included all records published from database inception to 12 November 2025, with no additional temporal restrictions.

A common core search strategy was applied across all databases, with minor adaptations to database-specific syntax when required. The search terms combined keywords related to speech and voice with those related to REM Sleep Behavior Disorder and biomarkers. The following Boolean expression was used: ((voice OR speech OR dysphonia) AND (REM Sleep Behavior Disorder OR RBD)) AND biomarker

All retrieved records were exported to Microsoft Excel 2021 (Microsoft Corporation, Redmond, WA, USA), where duplicate entries were identified and removed prior to screening. The same core search strategy was applied across databases with minor adaptations to database-specific syntax.

Eligibility Criteria

Eligibility criteria were defined a priori according to a PICOS-based framework and included additional specifications not detailed in the Introduction.

Inclusion Criteria

Studies were included if they met all of the following criteria:

- Population: adult human participants diagnosed with REM Sleep Behavior Disorder (idiopathic or secondary), based on clinical evaluation and/or polysomnographic confirmation.
- Exposure/Index Test: assessment of voice, speech, or language characteristics, including acoustic, prosodic, articulatory, or linguistic measures, collected during wakefulness and/or sleep.
- Comparator: healthy control participants and/or other clinical comparison groups, when available.
- Outcomes: quantitative or qualitative measures of vocal, speech, or language alterations; diagnostic, prognostic, or classification outcomes related to RBD or phenoconversion risk.
- Study Design: observational studies, including cross-sectional, longitudinal, case–control, or cohort studies.
- Time Frame: no restriction on the year of publication.
- Language: articles published in English.
- Publication Type: original research articles published in peer-reviewed journals.

Exclusion Criteria

Studies were excluded if they met any of the following criteria:

- Duplicate publications.
- Non-original research (systematic, narrative, or integrative reviews; meta-analyses; editorials; letters to the editor; conference abstracts; theses; gray literature).
- Studies not involving individuals with REM Sleep Behavior Disorder.
- Absence of objective voice, speech, or language assessment.
- Insufficient, incomplete, or incompatible data preventing extraction or synthesis.
- Single-case reports or studies with extremely small sample sizes.
- Publications not written in English.
- Studies with inadequate methodological quality, as identified during critical appraisal.

Reference lists of all included studies were manually screened to identify additional relevant publications.

### 2.2. Study Selection and Quality Assessment

The selection process involved three main phases. First, non-English articles and duplicates were removed. Then, titles and abstracts were screened for relevance and compliance with the inclusion and exclusion criteria. Finally, full-text versions of the remaining studies were reviewed in detail to confirm eligibility and ensure data completeness.

The quality of the studies included in this review was assessed using the Joanna Briggs Institute’s (JBI) checklists [41]. The systematic review was registered on OSF.io and is accessible at the following link: https://doi.org/10.17605/OSF.IO/QEJRH (accessed on 2 December 2025).

## 3. Results

**Figure 1:**
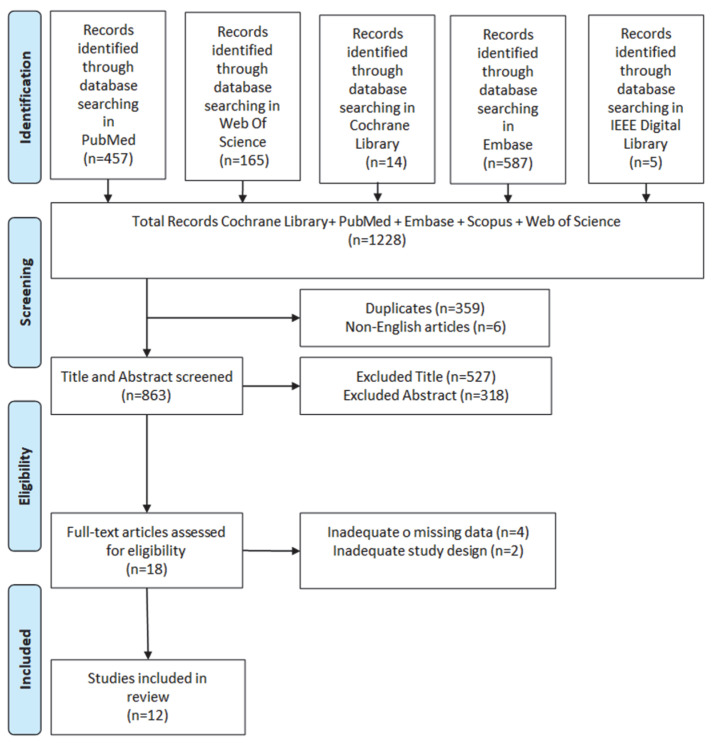
Process of study selection.

The electronic database search retrieved 1228 potentially relevant records. After duplicate removal, studies were screened based on title, abstract, and full-text evaluation. This process resulted in the inclusion of 12 eligible studies in the review (Figure 1).

Table 1 below provides a detailed overview of the 12 included studies. 

### 3.1. Quality Assessment Results

The methodological quality of the included studies was assessed using the Joanna Briggs Institute (JBI) critical appraisal checklists appropriate for each study design, specifically the cross-sectional analytical checklist and the cohort checklist. As summarized in Table 2 and Table 3, the overall methodological quality of the included studies was predominantly moderate, with higher quality observed in large-scale longitudinal cohort studies and multicenter studies. Limitations common to many studies included cross-sectional designs, small to moderate sample sizes, and incomplete control or adjustment for relevant confounding factors such as age, sex, language background, treatment status, and recording context. Although outcome measures were generally well defined and analytically appropriate, external validation and replicability were limited. These quality considerations indicate that the reported language, voice, and speech alterations should be interpreted as promising but preliminary, underscoring the need for larger, longitudinal, and methodologically harmonized studies to establish robust clinical biomarkers.

### 3.2. Overall Results

Across the twelve included studies [42,43,44,45,46,47,48,49,50,51,52,53], different research designs were employed, ranging from observational cross-sectional investigations to longitudinal cohort studies and multicenter digital phenotyping approaches. These studies were performed in several countries, including Germany, the Czech Republic, France, Japan, the United Kingdom, and multinational European collaborations. However, potential covariates such as age, sex, language background, medication status, comorbidities, and recording environment were not consistently controlled for or reported across studies, which may have influenced the observed speech and language outcomes. Despite methodological heterogeneity, all studies converged on analyzing speech, voice, or language-based features as potential biomarkers in idiopathic REM Sleep Behavior Disorder (iRBD), Parkinson’s disease (PD), or related synucleinopathies.

Regarding study aims, the earliest work focused on establishing whether nocturnal vocal events during REM sleep could predict neurodegeneration, whereas later studies shifted toward detailed acoustic, articulatory, or linguistic assessment of daytime speech. However, not all studies clearly differentiated between nocturnal REM-related vocalizations and daytime speech or language tasks, which may reflect partially distinct neurophysiological mechanisms and functional domains. More recent investigations extended this line of research by examining smartphone-based ecological speech or evaluating whether language alterations can forecast phenoconversion from iRBD to overt α-synucleinopathies.

The study populations varied from small samples of early PD or iRBD patients (*n* = 20–40) to larger single-center groups (*n* = 55–70) and multicenter multilingual cohorts. Some studies included both iRBD and early PD, whereas others compared patients with healthy controls or with NREM parasomnia cohorts. Although the inclusion of cohorts with early-stage PD has provided an important pathophysiological context, several studies have extrapolated findings from PD to iRBD, and the implications of combining or comparing these populations have not always been explicitly discussed. This limits the ability to distinguish prodromal features specific to iRBD from those reflecting established Parkinson’s disease pathology.

Speech-related tools used across studies included acoustic analyses of structured speech or connected speech, articulatory assessments, linguistic processing techniques, speech-graph analysis of dream reports, and automated extraction of speech parameters from natural smartphone recordings. However, differences in recording settings (e.g., laboratory-based, clinical, home, or smartphone-derived) were not systematically examined as potential sources of variability. Only a subset of studies relied on polysomnography, and when they did, they targeted vocal event scoring rather than general RBD diagnostic measures.

Speech and language tasks varied widely across studies, reflecting distinct methodological goals. Sustained vowel phonation, used by Rusz et al. [43] and Jeancolas et al. [50], assessed phonatory stability through jitter, shimmer, and HNR. Diadochokinetic “pa-ta-ka” sequences evaluated articulatory speed and regularity and were central in Rusz et al. [43,48]. Reading passages and fixed sentences offered controlled contexts for prosody, articulation, and timing analysis, as demonstrated by Polychronis et al. [46] and Hlavnička et al. [44]. Connected-speech monologs and spontaneous narratives enabled examination of lexical, syntactic, and semantic structure in studies by Šubert et al. [49,52] and Rusz et al. [48]. Dream-report narration provided access to REM-related narrative organization in See et al. [53]. Ecological smartphone call recordings captured real-world articulation and prosody in Illner et al. [51]. Finally, nocturnal speech during REM sleep, scored via polysomnography, documented shouting and speech-like utterances in Sixel-Döring et al. [42], Skorvanek et al. [45], and Takeuchi et al. [47], linking REM vocal behavior to early neurodegenerative progression. Despite this breadth, task-specific effects and their impact on outcome measures were not systematically compared across studies. Measures typically focused on articulation rate, speech rate, prosodic modulation, timing features, and phonatory stability (e.g., jitter, shimmer, HNR). Linguistic studies incorporated lexical diversity, syntactic complexity, semantic coherence, and graph-theoretical metrics derived from spontaneous narratives or dream reports. However, acoustic and linguistic features were often analyzed in parallel rather than within an integrated multimodal framework, limiting mechanistic interpretation across speech domains.

Results across studies consistently demonstrated that both daytime speech abnormalities and nocturnal vocal behaviors represent sensitive markers differentiating iRBD, early PD, and healthy controls. Since many studies were cross-sectional, involved small sample sizes, and had not undergone external validation or independent replication, diagnostic accuracy and prognostic significance should be interpreted with caution. Cross-sectional acoustic studies found that iRBD already presents with Parkinsonian-like alterations, such as reduced prosody, slowed timing, articulatory imprecision, and increased jitter or shimmer, similar to early PD. Linguistic analyses revealed significant impairments in lexical richness and syntactic organization. Furthermore, natural smartphone call recordings accurately detected early Parkinsonian changes in individuals with RBD. While several studies reported high sensitivity values, specificity, robustness across cohorts, and generalizability were less consistently examined. Longitudinal evidence confirmed that specific language features can predict phenoconversion from iRBD to neurodegenerative disease. Additionally, REM sleep vocalization frequency was identified as an early prognostic marker of motor progression in PD.

However, several limitations emerged across the included studies. Many investigations were cross-sectional and conducted with relatively small samples, limiting generalizability. Some studies relied solely on subjective vocal symptom reporting rather than objective acoustic metrics. Not all studies incorporated follow-up data, reducing insight into long-term predictive validity. In addition, the studies revealed considerable heterogeneity in terms of linguistic tasks, recording methods, languages, and analytical procedures. Studies using dream-speech or smartphone-derived data require replication, and variations in methodology (speech tasks, recording devices, analytic procedures) introduce challenges for cross-study comparison. Moreover, linguistic studies often lacked integration with acoustic measures, preventing a full multimodal understanding of speech alterations in RBD.

Overall, despite methodological differences, there is converging evidence that speech, voice, and language alterations, whether measured through acoustic parameters, articulatory performance, linguistic structure, or nocturnal vocal events, provide promising non-invasive biomarkers for early detection, monitoring, and prediction of neurodegenerative progression in idiopathic RBD.

### 3.3. Qualitative Analysis of Voice and Speech

Across studies adopting a qualitative or semi-structured descriptive approach, individuals with idiopathic REM sleep behavior disorder (iRBD) consistently showed alterations in vocal expression and speech behavior, both during REM sleep and wakefulness. Nocturnal vocalizations such as shouting, talking, and emotional utterances were frequently reported, as observed in Skorvanek et al. [45], where partner-reported dream-enactment vocalizations contributed to the clinical detection of RBD despite the absence of objective acoustic metrics. Similarly, Takeuchi et al. [47] found sex-related differences in REM vocal behavior among 55 iRBD patients, showing that women displayed fewer and less intense vocalizations than men, suggesting potential gender modulation of the disorder’s nocturnal vocal signature.

Beyond REM-related vocal behavior, qualitative language studies demonstrated meaningful disruptions in linguistic structure even in the absence of overt dysarthria. Šubert et al. [49] showed that individuals with iRBD produced spontaneous speech samples characterized by reduced lexical richness, simplified syntactic constructions, and diminished semantic coherence. These abnormalities were not attributable to cognitive decline, highlighting the presence of early linguistic disorganization. Extending this line of evidence, See et al. [53] compared dream-report speech from iRBD and NREM parasomnia patients and found that iRBD narratives were more linear and internally structured, suggesting that REM-related neurophysiology affects the organization of narrative speech content.

Qualitative findings across studies demonstrate that iRBD is characterized by (i) altered nocturnal vocal production, (ii) subtle but consistent impairments in expressive prosody and timing, and (iii) early disturbances in lexical, syntactic, and narrative structure. These abnormalities are detectable even when speech appears perceptually normal, underscoring the relevance of structured linguistic and voice assessments for early phenotyping.

### 3.4. Quantitative Analysis of Voice and Speech

Quantitative studies using objective acoustic, articulatory, or linguistic metrics consistently demonstrate measurable speech impairments in iRBD and early PD. Rusz et al. [43], studying 20 iRBD patients and 20 controls, reported significant articulatory and phonatory abnormalities, including impaired irregular alternating motion rates (*p* = 0.009), increased articulatory decay (*p* = 0.01), and elevated jitter and shimmer values (both *p* < 0.05). Their multifeature classification model achieved 96% sensitivity and 79% specificity for distinguishing iRBD from controls, suggesting strong diagnostic potential.

Using automated connected-speech analysis, Hlavnička et al. [44] examined 50 RBD patients, 30 early PD patients, and 50 controls. In this study was found that several timing- and prosody-related features, including rate of speech timing, pause duration, and entropy of timing, significantly differentiated RBD from controls (corrected *p* < 0.05). This model identified PD vs. controls with 71.3% accuracy, 56.7% sensitivity and 80.0% specificity for RBD cases with 70% accuracy, providing evidence that subtle acoustic anomalies emerge prior to motor symptoms.

Large-scale multilingual work by Rusz et al. [48] further quantified these deficits across 150 iRBD, 149 PD, and 149 control speakers. The composite dysarthria index was significantly abnormal in iRBD vs. controls (*p* = 0.002), with monopitch (*p* = 0.004) and pause frequency (*p* < 0.001) identified as robust discriminators. Over 12 months, speech impairment worsened in both iRBD (*p* = 0.04) and PD (*p* = 0.03), indicating measurable progression.

Jeancolas et al. [50] examined sustained vowels and connected speech in iRBD and early PD and found reduced pitch variability (*p* < 0.05) and phonation instability (elevated jitter/shimmer, *p* < 0.05) in iRBD relative to controls. These metrics closely matched those observed in early PD, reinforcing the concept of prodromal dysarthria.

Recent digital phenotyping findings further support the utility of quantitative vocal markers. Illner et al. [51] analyzed spontaneous smartphone call recordings from RBD patients and found that articulation precision and prosodic modulation significantly discriminated emerging parkinsonian signs, achieving AUC values > 0.70. Similarly, Šubert et al. [52] demonstrated that linguistic-timing and lexical-semantic metrics predicted phenoconversion from iRBD to Parkinson’s disease or other synucleinopathies with AUC = 0.80.

Taken together, quantitative results demonstrate that iRBD is consistently associated with measurable abnormalities in speech rate, articulation, prosody, phonation stability, pause structure, and linguistic output. These impairments appear across structured tasks, spontaneous speech, and nocturnal vocalizations, and in several studies, they show predictive value for future neurodegeneration.

## 4. Discussion

The purpose of this review was to examine whether vocal alterations observed during sleep and wakefulness could be considered exploratory, noninvasive candidate markers of REM sleep behavior disorder. Overall, the available evidence partially supports this objective, as several studies consistently report acoustic and linguistic abnormalities in individuals with RBD. However, given the substantial methodological heterogeneity and the limited availability of longitudinal validation, these findings should be interpreted as preliminary and indicative of exploratory, candidate biomarkers rather than clinically established biomarkers.

Vocal and Linguistic Alterations in RBD

Across twelve studies examining speech, voice, and language alterations in REM sleep behavior disorder, the evidence suggests a recurrent, though not uniform, pattern: individuals with RBD frequently exhibit measurable abnormalities in vocal production and linguistic organization that may precede the emergence of overt motor symptoms. The consistent detection of speech and language abnormalities across studies using different methods including acoustic analysis [43,48], articulatory measures [48], linguistic analysis [45], and ecological recordings [51] highlight the robustness of vocal alterations in iRBD. This convergence is notable given the cross-linguistic nature of the datasets, including multilingual cohorts, and suggests that the underlying mechanisms reflect shared neurophysiological processes rather than language-specific features [54].

Quantitative studies consistently indicate that speech abnormalities in RBD resemble the hypokinetic dysarthria characteristic of early Parkinson’s disease, including reduced pitch variability, slowed articulation, increased pauses, and phonatory instability [55,56]. These parallels reinforce the concept of iRBD as a prodromal stage of Parkinson’s disease and related disorders, where subtle bulbar motor abnormalities occur years before classic motor features [26]. However, these similarities do not imply diagnostic equivalence, and extrapolation from early Parkinson’s disease to iRBD should be interpreted with caution.

Recent linguistic studies revealed impairments in lexical selection, syntactic organization, semantic cohesion, and narrative structure in iRBD [49,53]. These abnormalities are not attributable to global cognitive deficits, suggesting early involvement of frontal and temporo-parietal language networks. Importantly, linguistic markers predicted phenoconversion with high accuracy, although replicability and external validation remain limited, underscoring the potential value of language analysis as a cognitive-linguistic biomarker [45,49,53].

Distinction Between Nocturnal and Daytime Vocal Phenomena

An important aspect that warrants explicit consideration is the distinction between nocturnal REM-related vocalizations and daytime speech and language abnormalities. Nocturnal vocalizations occur during REM sleep without atonia and primarily reflect dysfunction of brainstem circuits involved in REM-related motor inhibition [19]. Such vocal behaviors, including yelling, speech-like utterances, and emotionally charged vocal bursts, have been shown to predict clinical outcomes, including motor deterioration in early Parkinson’s disease [42].

In contrast, daytime speech and language abnormalities are assessed during wakefulness using structured or spontaneous tasks and are more likely to reflect early involvement of cortical, subcortical, and bulbar networks related to motor speech and linguistic processing [49,53]. Although both phenomena involve vocal output, they likely reflect partially distinct pathophysiological mechanisms and may differ in their value as biomarkers. Nocturnal vocalizations may provide disorder-specific information related to REM sleep circuitry, whereas daytime speech alterations may better capture generalized neurodegenerative processes and be more suitable for longitudinal monitoring. However, the current literature rarely examines these dimensions in a comparative or integrated manner.

Methodological Heterogeneity and Generalizability

The substantial heterogeneity observed across the included studies has important implications for both comparability and generalizability of the findings. Differences in study design (cross-sectional versus longitudinal), speech and language tasks (structured laboratory-based tasks, spontaneous speech, ecological recordings, and PSG-captured nocturnal vocalizations), and analytical pipelines limit the direct comparison of effect sizes and diagnostic performance across studies. Variability in recording conditions, feature extraction methods, and classification algorithms may lead to task-specific or context-dependent findings rather than a unified vocal phenotype of REM sleep behavior disorder [57,58].

Heterogeneity in study populations further constrains generalizability. Differences in age, disease duration, comorbidities, medication status, linguistic background, and risk of phenoconversion may influence vocal and linguistic measures, making it difficult to extrapolate results to the broader iRBD population. While cross-linguistic consistency suggests the involvement of shared neurophysiological mechanisms, the limited representation of certain languages, clinical subtypes, and female patients restricts external validity, underscoring the need for standardized protocols and multicenter designs [59,60].

Longitudinal Evidence and Prognostic Value

Only a limited subset of longitudinal studies directly evaluated phenoconversion, and most findings regarding disease progression are inferred from cross-sectional associations rather than demonstrated temporal change [48,52]. A critical appraisal of the available longitudinal studies highlights several limitations that constrain interpretation. Most investigations were conducted on relatively small cohorts, with heterogeneous follow-up durations and variable outcome definitions. In several cases, vocal or linguistic changes were assessed over time without direct confirmation of phenoconversion, limiting causal inference [26,52].

Moreover, differences in speech tasks, recording conditions, and analytical methods across longitudinal studies hinder direct comparison and replication. As a result, although longitudinal trends in vocal measures are suggestive of disease progression, current evidence remains insufficient to establish their predictive validity at the individual level.

Digital Phenotyping and Practical Challenges

The use of passive smartphone recordings represents a major advancement, enabling large-scale, low-burden, real-world assessment. Illner et al. demonstrated that articulation precision and prosodic modulation extracted from ordinary phone calls can detect subclinical Parkinsonism in iRBD [51]. These findings align with broader digital biomarker initiatives in neurodegeneration [61]. In this context, recent advances in digital sleep medicine have highlighted the growing role of consumer-grade technologies, including smartphones and wearable devices (e.g., smartwatches and fitness trackers), in sleep assessment and monitoring. These tools enable passive, longitudinal collection of speech, vocal, motor, and sleep-related data in ecologically valid environments. Smartphone-based voice recordings and wearable-derived sleep metrics have shown promising agreement with clinical assessments for detecting sleep fragmentation, abnormal vocalizations, and motor activity, supporting their potential utility for large-scale screening and longitudinal monitoring of sleep disorders, including RBD [39]. The integration of voice analytics with wearable sleep data may further enhance early detection strategies and facilitate remote monitoring in at-risk populations. Despite its promise, smartphone-based digital phenotyping faces several practical challenges that currently limit its translation into clinical practice. Variability in recording environments, background noise, microphone quality, and device hardware can substantially affect signal quality and feature extraction. Differences in user behavior, speech context, and long-term adherence introduce additional sources of variability that may confound longitudinal analyses. Furthermore, issues related to data privacy, ethical considerations, and regulatory requirements represent important barriers to large-scale implementation. The lack of standardized acquisition protocols, validated thresholds, and normative reference datasets further complicates result interpretation and increases the risk of false-positive or false-negative classifications [59,60,61].

Implications and Future Directions

Speech analysis offers several advantages over traditional biomarkers, including non-invasiveness, low cost, repeatability, and scalability. Such features make speech biomarkers particularly suitable for monitoring individuals with iRBD, who may remain asymptomatic for years despite a high risk of conversion. However, no single acoustic or linguistic feature has yet emerged as definitive.

Future research should prioritize large, multicenter longitudinal studies, standardized speech tasks and recording protocols, and integration with established prodromal markers such as REM sleep without atonia, olfactory loss, autonomic dysfunction, and dopamine transporter imaging [26,62]. Identifying a reproducible, disease-specific vocal signature validated across languages, devices, and clinical settings will be essential to advancing vocal analysis from an exploratory research tool to a clinically meaningful biomarker in RBD and related synucleinopathies.

### 4.1. Strengths and Limitations

One of the strengths of this work is the convergence of evidence gathered despite heterogeneity in study design. In addition, the inclusion of multilingual and multicenter cohorts strengthens the generalizability of language-related markers across different cultural and linguistic contexts. The main strength of this work lies in the integration of evidence on vocal alterations during both sleep and wakefulness and in the evaluation of voice-based measures that could, in the future, become scalable exploratory digital biomarkers suitable for longitudinal and remote monitoring. Furthermore, several studies have provided quantitative measures of diagnostic and predictive performance, offering preliminary evidence that language and speech alterations may serve as exploratory markers for early diagnosis and phenoconversion in synucleinopathies. Nevertheless, the current synthesis is limited and descriptive, as the heterogeneity of methodologies, outcome measures, and reporting standards across studies prevented a formal quantitative meta-analysis or direct comparison of effect sizes. Consequently, the robustness of the reported diagnostic accuracy metrics should be interpreted with caution. Additional limitations should also be acknowledged. Many studies were cross-sectional and involved small to moderate sample sizes, limiting statistical power and the ability to assess progression or causality. Longitudinal evidence remains limited, and only a few studies have directly linked linguistic or language characteristics to final phenotypic conversion. Language tasks and recording procedures varied considerably across studies, reducing comparability and complicating replicability. Some analyses relied in part or entirely on subjective reports of vocal behaviors, which may be influenced by patient or caregiver bias. Although linguistic abnormalities have emerged as a promising area, linguistic studies were scarce and often lacked integration with acoustic or articulatory measures, preventing a complete understanding of the mechanisms of language impairment. Moreover, although some studies reported high sensitivity or specificity, external validation and independent replication remain limited. Ecological approaches, including voice collection via smartphone, require further validation across different devices, environments, and patient subgroups. In the end, no single vocal or linguistic feature has yet achieved consensus as a definitive biomarker, underscoring the need for standardization.

### 4.2. Future Perspectives

The identification of a robust and reproducible vocal biomarker remains crucial for early diagnosis. Future research should prioritize large, multicenter longitudinal cohorts to validate speech and language measures and to establish their temporal stability. In addition, systematic control of relevant covariates, external validation, and integration within multimodal frameworks will be required before vocal measures can be considered clinically robust biomarkers. Standardization of language tasks, recording protocols, and analytical methods is essential to improve comparability across studies and to accelerate biomarker qualification. Integrating vocal characteristics with established prodromal markers, such as RSWA, olfaction, autonomic function, neuroimaging, and α-synuclein measures, will enable more accurate multimodal prediction models. In addition, digital phenotyping through passive smartphone recordings and home monitoring could offer powerful opportunities for real-world assessment. Last, further linguistic and narrative analyses could reveal early cognitive-linguistic signs [63,64].

## 5. Conclusions

Cumulative evidence from qualitative and quantitative investigations demonstrates that individuals with REM sleep behavior disorder exhibit consistent alterations in language, voice, and speech that extend to nocturnal vocalizations, structured linguistic activities, and spontaneous verbal production. This result directly addresses the primary objective of this review, which is to systematically identify and summarize studies investigating language, voice, and speech alterations in individuals with RBD. These abnormalities, although often subtle, are detectable with modern acoustic and linguistic analysis tools and appear to reflect early dysfunction in the neural circuits involved in synucleinopathies. In line with the second objective, the critical appraisal of the included studies revealed substantial methodological heterogeneity and variable risk of bias. Differences in study design, sample size, speech tasks, recording protocols, and analytical approaches limit the direct comparability of findings and underscore the exploratory nature of the current evidence base. With regard to the third objective, the descriptive comparison between studies indicates recurring, although not uniform, patterns of acoustic, prosodic, and linguistic anomalies. Concerning the objective relating to diagnostic and prognostic relevance, several studies show measurable differences compared to healthy controls and, in some cases, progressive changes or predictive accuracy for phenoconversion. These findings support the hypothesis that vocal and speech abnormalities may precede the emergence of overt motor symptoms and reflect early involvement of brainstem and cortico-subcortical networks implicated in α-synucleinopathies. As a result, the exploratory integration of vocal markers into clinical assessments appears promising but remains preliminary. Integrating these markers with established indicators such as polysomnography, olfactory testing, autonomic measures, and neuroimaging could significantly improve the sensitivity and specificity of early diagnosis. Furthermore, voice-based approaches offer a unique advantage due to their noninvasive, cost-effective, and scalable nature, allowing for frequent longitudinal monitoring in both clinical and real-world settings.

However, despite encouraging findings, no single vocal measure has yet achieved universal consensus as a standalone biomarker. The variability in tasks, metrics, and analytical procedures across studies underscores the need for continued systematic research aimed at identifying avalidating an exploratory, disease-relevant vocal signature. Establishing such a biomarker will require harmonized methodologies, large multicenter cohorts, and longitudinal designs capable of capturing trajectories of change. Advancing this line of investigation could ultimately yield a practical and sensitive tool for detecting prodromal neurodegeneration, stratifying risk, and monitoring treatment response in RBD and related disorders.

## Figures and Tables

**Table 1:** Studies included in the present review.

Reference	Type of Study	Country	Aim	Population	Tools	Tasks	Measures	Results	Limits
Sixel-Döring et al., 2014 [42]	Observational, longitudinal cohort	Germany	To determine whether REM vocal events predict neurodegeneration in early PD	20 early PD with REM behavioral events	Video-polysomnography scoring of REM vocalizations	REM sleep vocalizations	Frequency, duration, complexity of REM vocal events	Higher REM vocalization frequency predicted faster motor decline	Small sample; PD only; no daytime speech metrics
Rusz et al., 2016 [43]	Observational, cross-sectional	Czech Republic	Quantify motor speech abnormalities in idiopathic RBD	20 iRBD, 20 controls (*n* = 40)	Acoustic analysis of structured speech	Sustained vowels; Diadochokinetic tasks (AMR/SMR); Reading; Connected speech	Timing, articulation rate, prosody, jitter, shimmer, pausing	iRBD showed slowed timing, reduced prosody, articulation deficits, phonatory instability	Small sample; cross-sectional; no conversion follow-up
Hlavnička et al., 2017 [44]	Observational, cross-sectional	Czech Republic	Detect early vocal biomarkers in iRBD and early PD	12 iRBD, 12 early PD, 12 controls (*n* = 36)	Automated connected-speech analysis; acoustic processing	Connected speech monolog	Speech rate, articulation rate, prosody, jitter, shimmer, HNR	iRBD showed subtle PD-like speech abnormalities detectable with automation	Small sample; cross-sectional
Skorvanek et al., 2018 [45]	Observational cohort/screening validation	Slovakia, Czech Republic	Evaluate rating scales for RBD screening and prediction of conversion	67 iRBD, follow-up cohorts	Questionnaire vocal-behavior items	Partner-reported nocturnal vocalizations	Presence of dream-enactment vocalizations; subjective voice symptoms	Vocal symptoms improved detection but not conversion prediction	No objective acoustic data; subjective only
Polychronis et al., 2019 [46]	Observational, cross-sectional comparative	United Kingdom	Characterize speech impairments in early de novo PD	57 PD, 40 controls (*n* = 97)	Acoustic, articulatory speech analysis	Connected speech; Reading; Sustained vowels	Speech rate; articulation; pitch variability; jitter; shimmer	Early PD showed articulation deficits, reduced rate, prosodic flattening, phonatory instability	Not RBD; cannot infer prodromal RBD mechanisms
Takeuchi et al., 2020 [47]	Observational, cross-sectional	Japan	Examine sex differences in iRBD	55 iRBD	PSG scoring of REM vocalizations	REM sleep vocalizations	Frequency and intensity of REM vocal events	Women showed fewer and less intense REM vocalizations	No acoustic measures; nocturnal vocalizations only
Rusz et al., 2021 [48]	Observational, cross-sectional, multicenter	Multicenter	Identify universal speech biomarkers for iRBD and PD	Multi-language cohorts: iRBD, PD, controls	Standardized automated acoustic analysis	Reading; Monolog; Sustained vowels	Timing, articulation, prosody, phonation	Speech features reliably identified iRBD and PD across languages	Cross-sectional; no conversion data
Šubert et al., 2022 [49]	Observational, cross-sectional	Czech Republic	Identify linguistic abnormalities in iRBD	iRBD vs. controls	Linguistic analysis of spoken language	Spontaneous monolog	Lexical diversity; syntactic structure; semantic organization	iRBD had reduced lexical richness and altered syntax/semantics	No acoustic metrics; cross-sectional
Jeancolas et al., 2022 [50]	Observational, cross-sectional	France	Examine voice changes in iRBD to PD	iRBD, early PD, controls	Acoustic analysis of sustained vowels and speech	Sustained vowels; Connected speech	Pitch variability; intensity; timing; phonation stability	iRBD already showed PD-like acoustic deviations	Moderate sample; cross-sectional
Illner et al., 2024 [51]	Observational, digital phenotyping	Germany	Detect early parkinsonian markers from natural smartphone calls	RBD smartphone-recording cohort	Automated analysis of spontaneous phone speech	Natural ecological speech (phone calls)	Articulation precision; prosodic modulation; phonation	Natural speech predicted early parkinsonian signs better than clinical scales	Dependent on smartphone quality; needs replication
Šubert et al., 2024 [52]	Observational, multicenter, longitudinal	Multicenter (EU)	Test if language predicts phenoconversion in iRBD	iRBD converters vs. non-converters	Linguistic and speech sample analysis	Spontaneous monolog; Connected speech	Lexical, syntactic, semantic, timing measures	Baseline language alterations predicted conversion to synucleinopathy	Limited acoustic-only features; needs integration
See et al., 2024 [53]	Observational, retrospective	Australia	Compare dream-report speech in NREM parasomnia vs. iRBD	iRBD vs. NREM parasomnia	Speech-graph analysis of dream reports	Dream report narration	Graph-theoretical narrative metrics	iRBD produced more linear, structured dream speech	Indirect vocal marker; dream speech only

Legend: HNR = Harmonics to Noise Ratio; iRBD = Idiopathic Rapid Eye Movement Sleep Behavior Disorder; PD = Parkinson’s Disease; PSG = Polysomnography; RBD = Rapid Eye Movement Sleep Behavior Disorder; REM = Rapid Eye Movement.

**Table 2:** Methodological quality assessment of cross-sectional studies [42,43,44,45,46,47,48,50,53] investigating speech, voice, and language alterations in REM sleep behavior disorder, evaluated using the JBI Checklist.

Item JBI Checklist	JBI Quality Assessment Question	Study References								
		[42]	[43]	[44]	[45]	[46]	[47]	[49]	[50]	[53]
1	Were the criteria for inclusion in the sample clearly defined?	Yes	Yes	Yes	Yes	Yes	Yes	Yes	Yes	Yes
2	Were the study subjects and the setting described in detail?	Yes	Yes	Yes	Yes	Yes	Yes	Yes	Yes	Yes
3	Was the exposure measured in a valid and reliable way?	Unclear	Yes	Yes	Unclear	Yes	Yes	Yes	Yes	Yes
4	Were objective, standard criteria used for measurement of the condition?	Unclear	Yes	Yes	Unclear	Yes	Yes	Yes	Yes	Yes
5	Were confounding factors identified?	No	Unclear	Unclear	Unclear	Unclear	Unclear	Unclear	Unclear	Unclear
6	Were strategies to deal with confounding factors stated?	No	No	No	No	No	No	No	No	No
7	Were the outcomes measured in a valid and reliable way?	Unclear	Yes	Yes	Yes	Yes	Yes	Yes	Yes	Yes
8	Was appropriate statistical analysis used?	Yes	Yes	Yes	Yes	Yes	Yes	Yes	Yes	Yes

**Table 3:** Methodological quality assessment of longitudinal studies [48,51,52] examining vocal and speech-based markers in REM sleep behavior disorder and their prognostic relevance, evaluated using JBI Checklist.

Item JBI Checklist	JBI Quality Assessment Question	Study References		
		[48]	[51]	[52]
1	Were the two groups similar and recruited from the same population?	Yes	Yes	Yes
2	Were the exposures measured similarly to assign people to both exposed and unexposed groups?	Yes	Yes	Yes
3	Was exposure measured in a valid and reliable way?	Yes	Yes	Yes
4	Were confounding factors identified?	Unclear	Unclear	Unclear
5	Were strategies to deal with confounding factors stated?	No	No	No
6	Were the groups/participants free of the outcome at the start of the study?	Yes	Yes	Yes
7	Were the outcomes measured in a valid and reliable way?	Yes	Yes	Yes
8	Was the follow-up time reported and sufficient to be long enough for outcomes to occur?	Yes	Yes	Yes
9	Was follow-up complete, and if not, were the reasons described and explored?	Unclear	Unclear	Unclear
10	Were strategies to address incomplete follow-up utilized?	Unclear	Unclear	Unclear
11	Was appropriate statistical analysis used?	Yes	Yes	Yes

## Data Availability

No new data were created or analyzed in this study.

## Supplementary Materials

The following supporting information can be downloaded at: https://www.mdpi.com/article/10.3390/brainsci16020216/s1, PRISMA 2020 checklist.
